# Homer1a-Dependent Crosstalk Between NMDA and Metabotropic Glutamate Receptors in Mouse Neurons

**DOI:** 10.1371/journal.pone.0009755

**Published:** 2010-03-18

**Authors:** Federica Bertaso, Gautier Roussignol, Paul Worley, Joël Bockaert, Laurent Fagni, Fabrice Ango

**Affiliations:** 1 Departement of Neurobiology, Institut de Génomique Fonctionnelle, CNRS UMR 5203, INSERM U661, Université de Montpellier 1 & 2, Montpellier, France; 2 Department of Neuroscience, Johns Hopkins University School of Medicine, Baltimore, Maryland, United States of America; Medical College of Georgia, United States of America

## Abstract

**Background:**

A large number of evidences suggest that group-I metabotropic glutamate receptors (mGluR1a, 1b, 1c, 5a, 5b) can modulate NMDA receptor activity. Interestingly, a physical link exists between these receptors through a Homer-Shank multi-protein scaffold that can be disrupted by the immediate early gene, Homer1a. Whether such a versatile link supports functional crosstalk between the receptors is unknown.

**Methodology/Principal Findings:**

Here we used biochemical, electrophysiological and molecular biological approaches in cultured mouse cerebellar neurons to investigate this issue. We found that Homer1a or dominant negative Shank3 mutants that disrupt the physical link between the receptors allow inhibition of NMDA current by group-I mGluR agonist. This effect is antagonized by pertussis toxin, but not thapsigargin, suggesting the involvement of a G protein, but not intracellular calcium stores. Also, this effect is voltage-sensitive, being present at negative, but not positive membrane potentials. In the presence of DHPG, an apparent NMDA “tail current” was evoked by large pulse depolarization, only in neurons transfected with Homer1a. Co-immunoprecipitation experiments showed interaction between G-protein βγ subunits and NMDA receptor in the presence of Homer1a and group-I mGluR agonist.

**Conclusions/Significance:**

Altogether these results suggest a direct inhibition of NMDA receptor-channel by Gbetagamma subunits, following disruption of the Homer-Shank3 complex by the immediate early gene Homer1a. This study provides a new molecular mechanism by which group-I mGluRs could dynamically regulate NMDA receptor function.

## Introduction

The neurotransmitter glutamate activates both ionotropic (AMPA, kainate and NMDA subtypes) and metabotropic (mGluR1-8 subtypes) receptors at mammalian central synapses. The AMPA and kainate receptor subtypes are responsible for fast post-synaptic responses, while NMDA receptors (NMDA-Rs) mediate long-term synaptic plasticity and neurotoxicity. Among the eight mGluR subtypes, mGluR1 and mGluR5 (group-I mGluRs) are localized in an annulus that circumscribes the postsynaptic density (PSD) [1]. As they display low affinity for glutamate, optimal activation of these receptors would be achieved only upon large synaptic release of the neurotransmitter glutamate.

Crosstalk between group-I mGluRs and NMDA-Rs has long been investigated by examining the effect of prestimulation of the mGluRs on subsequent evoked NMDA currents. The majority of these studies have demonstrated a facilitatory effect [2], although inhibitory effects have also been reported in organotypic hippocampal slices [3]. It is worth noting that because of localization of NMDA-Rs within the PSD and group-I mGluRs at its edge, synaptically released glutamate should activate either NMDA-Rs solely, or both NMDA-Rs and group-I mGluRs concomitantly, rather than group-I mGluRs first and NMDA-Rs subsequently. It is therefore relevant to investigate the functional consequence of strict co-activation of the NMDA-Rs and group-I mGluRs.

The Shank proteins (Shank1, Shank2 and Shank3) form a large multimeric complex at the base of the PSD and co-assemble group-I mGluR1a/5 with NMDA-Rs through the dimeric adaptor proteins, Homer (Homer1b, Homer1c, Homer2 and Homer3, here referred to as Homer c-c for Homer containing a coiled-coil domain) and the GKAP-PSD95 protein complex respectively [4]. The immediate early gene, *homer1a*, is induced in an activity-dependent manner and the product of this gene lacks the coiled-coil domain. Thus, it acts as a dominant negative monomeric regulator to antagonize the interaction between constitutively expressed Homer c-c and group-I mGluRs, in response to elevated neuronal activity [5]. In the present study we examined whether this versatile multiprotein complex could underlie a functional crosstalk between mGluR1a and NMDA-Rs when these receptors are strictly co-activated, in cultured neurons. We found no crosstalk between these receptors under control condition, but potent inhibition of NMDA currents by mGluR1a following physical disruption of the mGluR1a/Homer c-c/Shank3 complex.

## Results

### Homer1a allows inhibition of NMDA-Rs by mGluR1a

**Figure 1:**
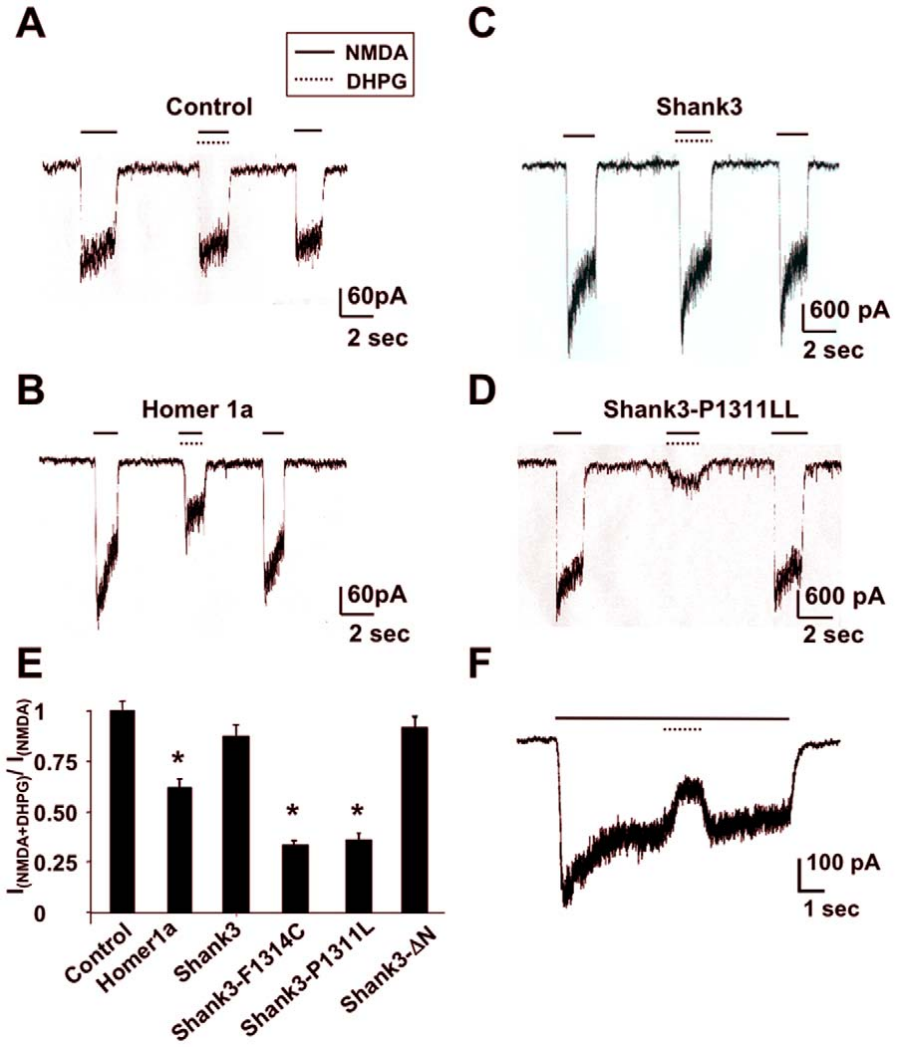
Inhibition of NMDA currents after disruption of the mGluR1a-Homer-Shank3 complex. **A–D**: NMDA currents recorded in control neurons (A), in neurons transfected with Homer1a (B), Shank3 wild-type (C), or the indicated Shank3 point mutant (D). **E**: The inhibitory effect of DHPG was quantified by plotting the amplitude of the current evoked by co-application of NMDA and DHPG over the amplitude of the current evoked by NMDA alone, in the same cell. Each value of the histogram is the mean (± s.e.m.) of 10 experiments. **F**: A brief application of DHPG was performed during the application of NMDA, in a Homer1a transfected neuron, showing fast onset and reversibility of its inhibitory effect on NMDA current. Similar results were observed in all the recorded neurons (n = 10). In this and the following figures, all the currents were recorded at a holding potential of −80 mV.

Primary cultures of cerebellar granule neurons express both mGluR1a (but not mGluR5) and NMDA-Rs [6]. We first examined whether co-activation of these receptors has a consequence on the NMDA current in these cells, using whole-cell patch-clamp recording. Co-application of NMDA and DHPG, a selective group-I mGluR agonist, elicited a significant and virtually immediate inhibition of the NMDA currents in neurons transfected with Homer1a (Fig. 1B, E), but not in non-transfected neurons (Fig. 1A, E). A transient exposure of the neurons to DHPG, during application of NMDA, also blocked the NMDA currents in less than 500 msec (Fig. 1F). These effects were rapidly and fully reversible upon washout of DHPG (Fig. 1A, B, F). No effect of DHPG was observed on AMPA- (50 µM) or muscimol- (GABA_A_ agonist; 10 µM) induced currents, in neurons expressing Homer1a (data not shown). We sought to determine whether the NMDA current inhibition is solely dependent on Homer1a, or rather due to a more global effect on the scaffolding complex. Similar experiments were performed in neurons transfected with dominant negative point mutants, Shank3-P1311L or Shank3-F1314C, which do not bind Homer proteins [4]. The Shank3 mutants, but not Shank3 wild-type, also allowed inhibition of NMDA currents by DHPG in the transfected neurons (Fig. 1C, D, E). It should be noted that the large amplitude of NMDA currents in panels C and D, in the absence of DHPG, likely resulted from the recruitment of functional NMDA receptors by Shank3 (wild-type and mutant) [7]. These results suggested implication of the Homer-Shank3 complex dissociation, rather than an effect of Homer1a *per se*, in the inhibition of NMDA-R by mGluR1a, when both receptors were concomitantly activated.

Interestingly, DHPG-induced inhibition of NMDA current was not observed in neurons transfected with a Shank3 mutant (Shank3-ΔN) that does not bind to the GKAP-PSD95-NMDA-R complex (Fig. 1E). This indicated that inhibition of NMDA current by mGluR1a depends selectively on the Homer, but not GKAP interaction with Shank3.

### The inhibitory crosstalk between NMDA-R and mGluR1a is G protein- and voltage-dependent, but Ca^2+^-independent

**Figure 2:**
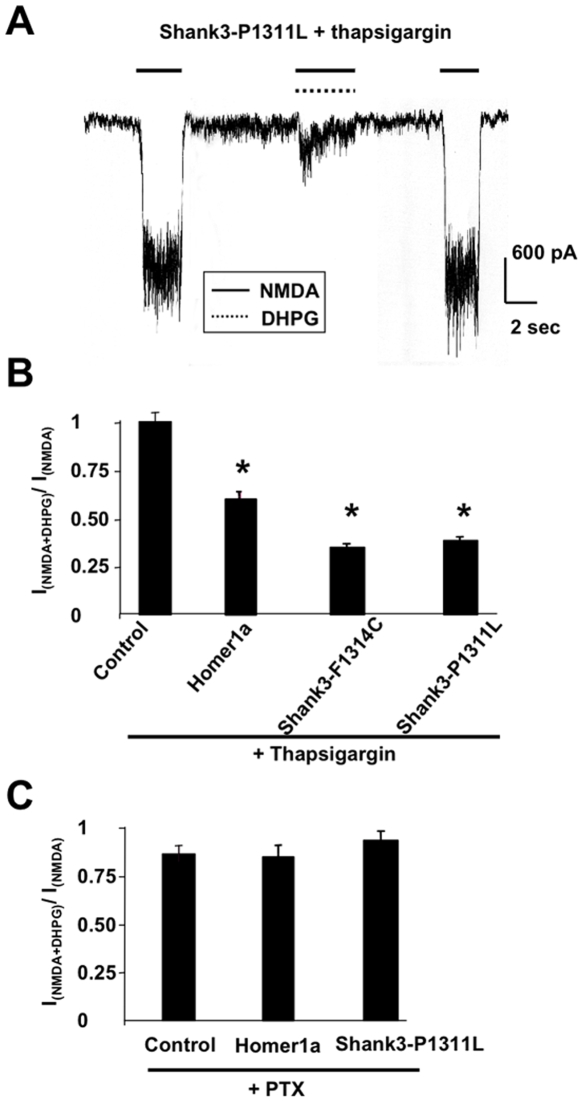
Crosstalk between NMDA-R and mGluR is dependent on G-protein activation, but independent of intracellular Ca^2+^ release. **A**: NMDA currents recorded in a neuron transfected with the indicated Shank3 mutant and pre-treated for 30 min with thapsigargin. **B**: Quantification of the inhibitory effect of DHPG on NMDA currents as described in Fig. 1E, in control neurons and neurons transfected with the indicated plasmids, and treated with thapsigargin. **C**: Same legend as in B, but in neurons pre-treated with PTX.

We further examined the mechanisms of this receptor crosstalk in neurons expressing Homer 1a. Depletion of intracellular Ca^2+^ stores with thapsigargin (1 µM, 30 min) did not alter the inhibitory effect of DHPG on NMDA currents (Fig. 2A, B), suggesting that mobilization of intracellular Ca^2+^ stores by mGluR1a was not required.

In cultured cerebellar granule cells, mGluR1a is coupled to a pertussis toxin (PTX)-sensitive G protein [8]. Pre-treatment of the neurons with the toxin abolished the negative crosstalk between mGluR1a and NMDA-R (Fig. 2C), suggesting that the effect was G protein-mediated. Qualitatively similar results were observed in neurons transfected with the recombinant Shank3-P1311L or Shank3-F1314C mutants (Fig. 2A, B, C).

**Figure 3:**
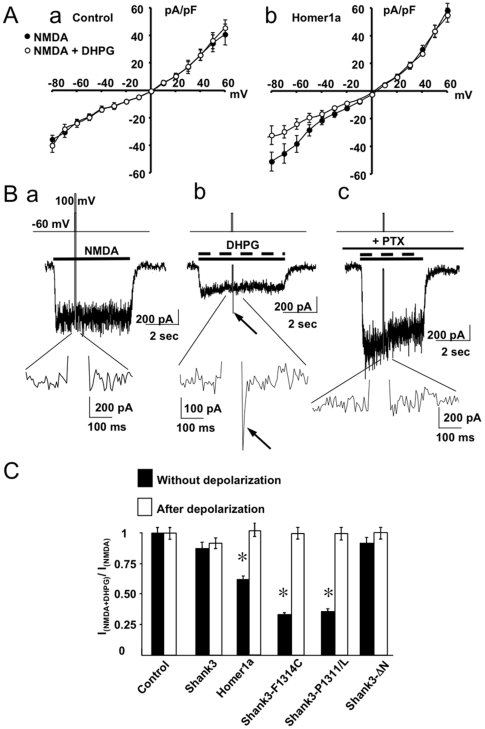
Voltage-dependency of NMDA-R and mGluR crosstalk. **A**: Current-voltage relationships obtained with applications of NMDA alone (filled circles) or co-applications of NMDA and DHPG (open circles), in control neurons (a) and neurons transfected with Homer1a (b). **B**: NMDA currents recorded in neurons transfected with Homer1a. The upper traces illustrate the voltage protocol applied to the neurons. Each lower trace is the expanded part of the middle trace (current) during the depolarizing pulse. The arrow in b indicates the NMDA “tail current”. Panels a and b were obtained in the absence of PTX treatment, while panel c was obtained in a PTX treated neuron. **C**: Quantification of the inhibitory effect of DHPG on NMDA current recorded before (filled bars; identical to those illustrated in Fig. 1E) and immediately after (open bars; NMDA “tail current” measured from baseline ( = 0 pA current)) the depolarization pulse, as described in Fig. 1E. Each value of the histogram is the mean (± s.e.m.) of at least 10 experiments.

NMDA current-voltage relationships revealed that co-applied DHPG inhibited NMDA currents only at membrane potentials ranging from −100 to −40 mV, in neurons expressing Homer1a, but not in control neurons (Fig. 3A). This indicated a voltage-dependency of the inhibitory effect of mGluR1 on NMDA channels in the presence of Homer1a.

### Inhibition of NMDA current by mGluR1a through direct interaction of Gβγ subunits with NMDA-R

The rapid kinetics, together with G protein- and voltage-dependency of NMDA current inhibition by mGluR1a were reminiscent of direct inhibition of voltage-sensitive N- and P/Q-type Ca^2+^ channels by Gβγ subunits [9]. Inhibition of the Ca^2+^ channels by Gβγ subunits can be reversed by elevated (up to 100 mV) transient membrane depolarization. Similarly, 100 msec depolarization to +100 mV applied during co-application of NMDA and DHPG evoked an apparent NMDA “tail current” after cessation of the depolarizing pulse, in neurons transfected with Homer1a (Fig. 3Bb) or Shank3-F1314C and Shank3-P1311L mutants, but not in control neurons (Fig. 3C). No “tail current” was observed in neurons expressing Homer1a, in the absence of DHPG (Fig. 3Ba). It is worth noting that the amplitude of the NMDA “tail current” was not significantly different from that of NMDA current recorded in control neurons (in the absence of Homer1a and DHPG; ratio value of 1 in Fig. 3C). A PTX treatment abolished the NMDA “tail current” (Fig. 3Bc). These results suggested that the NMDA “tail current” corresponded to residual transient desinhibition of NMDA channels by Gβγ, after cessation of the depolarization pulse. No “tail current” was observed on responses evoked by AMPA or muscimol upon co-application of DHPG in the presence of Homer 1a, Shank3-F1314C or Shank3-P1311L mutants (data not shown), suggesting that the effect was specific to NMDA channels.

No NMDA “tail current” was observed in neurons transfected with the Shank3-ΔN mutant (Fig. 3C). This observation further supported our above hypothesis that the G protein-mediated inhibition of NMDA channels by DHPG required disruption of the mGluR1a/Homer c-c/Shank3, but not NMDA-R/PSD-95/GKAP complex.

**Figure 4:**
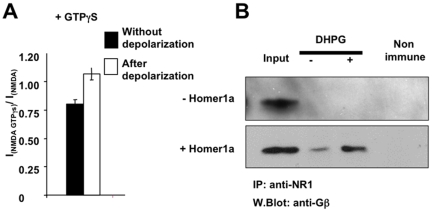
G-proteins interact with and inhibit NMDA channels. **A**: GTPgammaS was dialyzed in cultured cerebellar granule cells through the recording pipette and NMDA currents measured before and after a depolarization pulse, as described in Fig. 3Ba and Fig. 3C. **B**: Control neurons (- Homer1a) or neurons expressing induced Homer1a (+ Homer1a) were exposed (+) or not (−) to DHPG, plus NMDA. Lysates were prepared from these cultures and immunoprecipitated with a pan anti-NR1 antibody, and revealed with an anti-Gbeta antibody.

To confirm the action of G proteins on NMDA channel, GTPγS (100 µM), a drug that activates G proteins in a non-selective manner, was dialyzed into neurons. The density of NMDA current was statistically smaller in these neurons than in non-dialyzed neurons. Moreover, transient depolarization allowed expression of a NMDA “tail current”, even in the absence of Homer1a and DHPG. Once more, the peak amplitude of this “tail current” was not significantly different from the amplitude of the NMDA current obtained in the absence of GTPγS (Fig. 4A). Neither GDPβS nor GTP affected NMDA currents (data not shown). Finally, co-immunoprecipitation experiments were performed in control neurons and in neurons expressing induced Homer1a, in the absence and presence of DHPG. The immunoblots showed that in the presence of DHPG, Gβ-containing subunit could be co-immunoprecipitated with the NR1 subunit only when Homer1a was induced (Fig. 4B). This provided further evidence for formation of a complex between Gβγ and NMDA-Rs. In the absence of DHPG, only a faint band of Gβ was co-immunoprecipitated with NR1, probably due to a weak stimulation of mGluR1a by endogenously released glutamate and slight association of Gβγ with NMDA-R.

## Discussion

In the present study we examined whether the physical link established between group-I mGluRs and NMDA-R by the Shank3 multiprotein complex could be responsible for a functional crosstalk between these two receptors. We found that disruption of the link between Shank3 and the Homer-mGluR1a/mGluR5, but not GKAP/PSD-95/NMDA-R complex, allows direct binding of the activated Gbetagamma subunits to the NMDA channel, upon concomitant activation of the group-I mGluRs, and rapid inhibition of the channel.

While Gbetagamma binding to voltage-dependent channels (VDCC) is well documented [9], interaction of G protein subunits to ligand-gated ion channels is an emerging issue. For instance, G proteins increase the activity of glycine and acetylcholine receptors. Conversely, in cortical neurons, NMDA currents were reduced upon application of group-I agonists. Similar to our results, the authors found that intracellular Ca^2+^ was not involved in the inhibitory signalling cascade, whereas GTPgammaS could mimic and PTX could block the effect. However, no further direct evidence for G-protein modulation of NMDA receptors was provided [10], [11], [12]. Here we show that Gbetagamma subunits co-immunoprecipitate with the NMDA receptor complex in neuronal tissue.

GPCR-mediated inhibition of VDCC is based on the interaction between the Gbetagamma subunit and the pore-forming alpha subunit of the channel. It is however the accessory beta subunit that mediates the voltage-dependence of the inhibition [13]. Intramolecular movements of the alpha subunits, notably the S6 transmembrane segment, would cause the release of Gbetagamma from the channel. This suggests that only the reversal of inhibition of the channel, but not the inhibition itself, involves a voltage-dependent mechanism. Consistent with this hypothesis, Gbetagamma could inhibit the NMDA receptor despite its lack of canonical voltage sensor directly responsive to membrane potential variations. The reversal of inhibition that we observed upon depolarization might be due to a different mechanism. One can tentatively suggest that Gbetagamma unbinding to the receptor would depend on electrostatic charges on particular portions of the protein, which would be sensitive to changes in membrane polarization. A hallmark of Gβγ binding motifs of ion channels and receptors is the presence of basic residues [9], [14]. Interestingly, *in-silico* analysis of amino-acid sequences of NMDA-R subunits revealed a potential stretch of basic residues in the first intracellular loop of NR2C (unpublished results). Interestingly, this subunit is expressed in cerebellar granule cells (Figure S1). Nevertheless, no basic residue has been found to be important for Gbetagamma regulation of GIRK channels [15]. Therefore, further studies are required to validate the nature of the NMDA subunit that is recognized by Gbetagamma subunits.

How could Homer1a allow functional inhibitory crosstalk between NMDA-R and mGluR1a? One possibility is that the competitive action of Homer1a on Homer c-c binding sites would isolate mGluR1a from the multiprotein Shank3 complex, thus allowing lateral translocation of the receptor towards the PSD. This would bring mGluR1a and NMDA-Rs into close vicinity and facilitate membrane-delimited interaction of mGluR1a-activated G-protein with the NMDA channel. Consistent with this hypothesis, we found that Shank3 mutants that do not bind to Homer proteins also allow inhibition of NMDA current by mGluR1 agonist. Previous observations also support this model. Group-I mGluRs display a high degree of membrane confinement when interacting with constitutively expressed Homer c-c proteins, but lose such a confinement when binding to Homer1a [16]. In addition, Homer c-c proteins support mGluR1 clustering and prevent inhibition of N-type Ca^2+^ and M-type K^+^ channels by mGluR1. Upon Homer1a expression, mGluR1 becomes uniformly distributed on the cell surface and triggers inhibition of these channels in a Gbetagamma-dependent manner [17].

Crosstalk between NMDA-Rs and Group-I mGluRs is controversial. Some studies show up-regulation [2] while others found down-regulation [12] of NMDA-R functions by group-I mGluRs. Due to the variety of experimental paradigms used in these studies, no straightforward conclusion can be drawn. However reminiscent to our data, Yu *et al*. [12] found an inhibitory crosstalk between NMDA-Rs and Group-I mGluRs in neurons, which was G-protein dependent and membrane delimited. None of these studies examined the role of the Shank complex in the crosstalk.

To conclude, we provide evidence that Homer1a may allow a fast and reversible negative feedback control of NMDA-R functions via group-I mGluRs. Although the patho-physiological significance of this crosstalk remains to be elucidated, we tentatively propose that it could regulate synaptic plasticity and/or excitotoxicity as NMDA-Rs play a crucial role in these phenomena. Such crosstalk could also contribute to psychiatric disorders, since a Shank3 deletion mutant lacking the Homer binding site has been identified in autism [18].

## Methods

### Ethics statement

All animal work was conducted in strict accordance to the rules and regulations of the French animal welfare bodies and the French Ministry for Industry and Agriculture.

### Culture preparation and transfection

Primary cultures of cerebellar neurons were prepared from postnatal day 6–8 mice as previously described [19]. Neurons were dissociated and plated in 35 mm diameter Petri dish coated with poly-L-ornithin at a density of 3−5×10^5^ cells/dish. Neurons were transfected immediately before plating with Shank3 wild-type or Shank3 mutants cDNA expression plasmids [7] using a previously described lipofection method [6]. To block G protein activation, cerebellar cultures were treated overnight with PTX (200 ng/ml). Experiments were performed from 10–12 DIV mature neurons and data collected from at least three different dishes, from at least three different cultures.

### Electrophysiology

Cerebellar granule cells were recorded at room temperature using the whole-cell configuration of the patch-clamp technique as previously described [7]. Agonists were supplied to this medium and applied using a fast gravity perfusion system that allowed complete exchange of the cell environment in less than 30 ms [20]. Currents were recorded using an Axopatch 200B amplifier (Axon Instruments, Molecular Device Corp., Sunnyvale, CA, USA), filtered at 1 KHz and stored on a tape recorder and/or a PC. In experiments using the +100 mV depolarizing pulse (100 msec duration), records were leak subtracted using the PCLAMP8 P/20 routine. NMDA-mediated currents were measured at their peak amplitude and data expressed as mean ± s.e.m. Statistical differences between groups were tested using the non-parametric Wilcoxon's test. They were considered significant at p≤0.05 (*).

Drug concentrations and suppliers were as follows: NMDA (100 µM; Sigma, France), DHPG (100 µM; Tocris), muscimol (10 µM; Sigma), AMPA (50 µM, Sigma), GTPgammaS (100 µM; Sigma), thapsigargin (10 µM; Sigma), PTX (200 ng/ml final concentration; Sigma).

### Biochemical analyses

For co-immunoprecipitation experiments, lysates prepared from 10–12 DIV cerebellar cultures. Homer1a was induced using co-application of NMDA and kainate (100 µM each) for 1 hour and then treated with MK801 for 6 hours, as previously described [19]. Immunoprecipitation was performed in these neurons after treatment with either NMDA or NMDA + DHPG using a polyclonal anti-NR1 antibody (Zymed, CA, USA). Western blots were performed using an anti-Gbeta antibody (1/1000 dilution) as primary antibody and an anti-IgM secondary antibody (1/10000).

## Supporting Information

Figure S1 Western blot showing the expression of both NR2A/B and NR2C subunits in cerebellar granule cell culture extract.
(3.82 MB TIF)
