# Supplementary figures and images for: Homer1a-Dependent Crosstalk Between NMDA and Metabotropic Glutamate Receptors in Mouse Neurons
Source: PLoS One. 2010 Mar 18;5(3):e9755. doi: 10.1371/journal.pone.0009755 (PMC2841198; doi:10.1371/journal.pone.0009755)

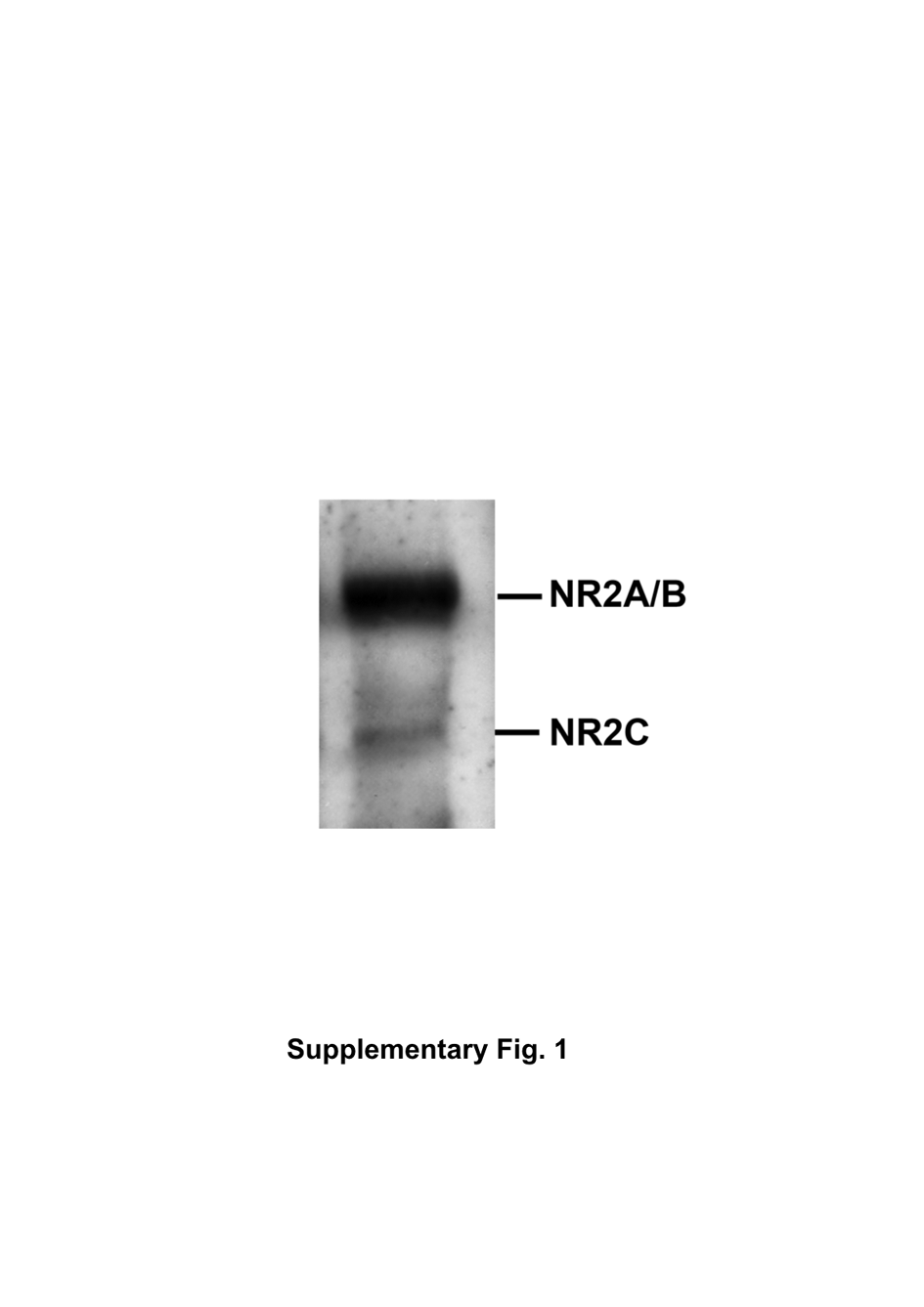

Supplement: Figure S1 — Western blot showing the expression of both NR2A/B and NR2C subunits in cerebellar granule cell culture extract. (3.82 MB TIF) [file pone.0009755.s001.tif]
